# Simultaneous Resection of Pancreatic Neuroendocrine Tumors with Synchronous Liver Metastases: Safety and Oncological Efficacy

**DOI:** 10.3390/cancers14030727

**Published:** 2022-01-30

**Authors:** Pietro Addeo, Caterina Cusumano, Bernard Goichot, Martina Guerra, François Faitot, Alessio Imperiale, Philippe Bachellier

**Affiliations:** 1 Hepato-Pancreato-Biliary Surgery and Liver Transplantation, Pôle des Pathologies Digestives, Hépatiques et de la Transplantation, Hôpital de Hautepierre-Hôpitaux Universitaires de Strasbourg, Université de Strasbourg, 67098 Strasbourg, France; caterina.cusumano@chru-strasbourg.fr (C.C.); martina.guerra@chru-strasbourg.fr (M.G.); francois.faitot@chru-strasbourg.fr (F.F.); philippe.bachellier@chru-strasbourg.fr (P.B.); 2 Internal Medicine, Diabetes and Metabolic Disorders, University Hospitals of Strasbourg, Strasbourg University, 67200 Strasbourg, France; bernard.goichot@chru-strasbourg.fr; 3 Nuclear Medicine and Molecular Imaging, Institut de Cancérologie de Strasbourg Europe (ICANS), University Hospitals of Strasbourg, Strasbourg University, 67200 Strasbourg, France; a.imperiale@icans.eu; 4 Molecular Imaging—DRHIM, IPHC, UMR 7178, CNRS/Unistra, 67037 Strasbourg, France

**Keywords:** neuroendocrine tumors, liver metastases, liver resection, pancreatic resection, survival

## Abstract

**Simple Summary:**

Up to half of all newly diagnosed pancreatic neuroendocrine tumors (PNET) present with liver metastases (LM). The surgical resection of PNETs and LMs can provide complete tumor clearance and improve long-term survival. However, the combination of liver and pancreatic resection simultaneously can theoretically cumulate the morbidity and mortality of two separate operations. In the current study, we analyzed the outcomes of the synchronous surgical resection of PNETs and LMs in 51 patients. There were no differences in the postoperative outcomes in terms of mortality and morbidity according to the type of pancreatic resection. The tumor grade was identified as the sole prognostic factor for survival. The resection of well-differentiated PNETs with LMs was characterized by the longest survival rates (median overall survival 128 months, 5-year overall survival 83%). The optimal sequential surgical strategies for PNETs with LM and the use of neoadjuvant/adjuvant chemotherapy in this category of patients remain to be further investigated.

**Abstract:**

Whether the simultaneous resection of pancreatic neuroendocrine tumors (PNET) with synchronous liver metastases (LM) is safe and oncologically efficacious remains to be debated. We retrospectively reviewed clinical data from patients who underwent the simultaneous resection of PNETs with LMs over the last 25 years. Fifty-one consecutive patients with a median age of 54 years (range 27–80 years) underwent pancreaticoduodenectomy (PD) (*n* = 16), distal pancreatosplenectomy (DSP) (*n* = 32) or total pancreatectomy (*n* = 3) with synchronous LM resection. There were no differences in the postoperative outcomes in term of mortality (*p* = 0.33) and morbidity (*p* = 0.76) between PD and DSP. The median overall survival (OS) was 64.78 months (95% CI: 49.7–119.8), and the overall survival rates at 1, 3, and 5 years were 97.9%, 86.2% and 61%, respectively. The OS varied according to the tumor grade (G): G1 (OS 128 months, 5-year OS 83%) vs. G2 (OS 60.5 months, 5-year OS 58%) vs. G3 (OS 49.7 months, 5-year OS 0%) (*p* = 0.03). Multivariate Cox analysis identified G as the only prognostic factor (HR: 5.56; 95% CI: 0.91–9.60; *p* = 0.01). Simultaneous PNETS with LMs can be performed safely with acceptable morbidity and mortality at tertiary centers. Well-differentiated PNETs had longer survival and might benefit the most from these extended surgeries.

## 1. Introduction

Pancreatic neuroendocrine tumors (PNET) are a rare neoplasm with a biological behavior ranging from indolent to highly aggressive disease. The therapeutic management is based on tumor, disease and patients factors [1,2]. Up to half of all newly diagnosed PNETs present with liver metastases (LM) [3]. The direct venous drainage of the pancreas into the portal system and the tendency of PNETs to spread through tumor thrombosis might explain the high predisposition of PNETs to metastasize to the liver [4].

The treatment of liver metastases of PNET ranges from surgical resection to palliative chemotherapy according to the resecability of the liver metastases. In the presence of resectable disease, usually with less than 50% of the liver volume occupied by LMs, surgery can provide complete tumor clearance and improve long-term survival [1,5]. However, recurrence after the resection of liver PNETs remains expected, but when limited to the liver, it can benefit from repeat local and systemic therapy [3]. Primary PNETs with synchronous resectable liver metastases might pose a surgical challenge, as combining liver and pancreatic resection could add up the morbidity and mortality of two separate potentially highly morbid operations. This could be especially the case for synchronous pancreaticoduodenectomy with major liver resection, which has been characterized by a postoperative mortality rate up to 17% [6]. Several surgical strategies have been reported in presence of synchronous LMS and PNETs, including primary tumor resection and staged liver resection, and the synchronous resection of PNET and LM [7,8]. Because of the rarity of the disease and the need for a combined expertise in liver and pancreatic surgery, series analyzing the outcomes of the synchronous resection of pancreatic PNET with LM remain limited [5,7,9,10,11,12]. Herein, we analyze our overall experience in the resection of pancreatic neuroendocrine tumors with synchronous liver metastases, evaluating the safety and oncological efficacy of these combined resections. 

## 2. Materials and Methods

This retrospective cohort study was conducted at the Hepato-Pancreato-Biliary Surgery and Liver Transplantation Center of the University of Strasbourg in France. The study was conducted according to the guidelines of the Declaration of Helsinki. Data were retrospectively analyzed for all of the patients who underwent pancreatic resection for PNET in presence of LM between 1 January 1995 and 31 December 2020. The collected data included demographic, operative (operative time, transfusion requirement, 90-day morbidity and mortality rates), and survival variables. Details were collected on the type of vascular resections (venous and arterial) and the presence and type of the associated visceral resection (colonic, gastric, and intestinal). The preoperative evaluation included thoraco-abdominal computed tomography and/or the magnetic resonance imaging of the abdomen. The functional imaging included OctreoScan In 111 and then PET-Ga 68 when this became available to our institution. Surgical resection was scheduled for patients with good performance status in whom the complete surgical resection of pNET and LMs was judged to be feasible in one-stage or two-stage procedures. The treatment of the LMs included conventional resection and radiofrequency (RF) ablation, and/or a combination of the two. The extent of the pancreatic resection included right resections [(pancreaticoduodenectomy (PD), total pancreatectomy (TP), and distal spleno-pancreatectomy (DSP)]. Major liver resections were defined as the resection of three or more contiguous liver segments. Morbidity was graded according to the Dindo-Clavien classification, with major morbidity including complications classified as IIIA or above [13]. Pancreas-specific complications were defined according to the recommendations from the International Study Group for Pancreatic Surgery, including pancreatic fistula, delayed gastric emptying, and post pancreatectomy hemorrhage [14,15,16]. PNETs were classified according to the Ki67 index into 3 categories: PNET G1 (<3), PNET G2 (3–20), and PNET G3 (>20) [1]. One expert pathologist reviewed all of the specimens for KI-67 assessment. Follow-up visits were regularly completed in the outpatient clinic and/or with the referring clinicians. OS was defined as the length of time from the date of surgery to the patient’s death or the last follow-up. The date of the last follow-up was 30 June 2021.

### Statistical Analysis

The continuous variables are expressed as means ± standard deviations or medians and ranges, as appropriate, and the categorical variables are presented as numbers and percentages. The differences between the groups were assessed by the chi-squared or Fisher’s exact test for categorical variables, as appropriate. In order to compare the continuous variables, the Wilcoxon rank-sum test and Student’s *t*-test were used, as appropriate. The survival estimates were calculated according to the Kaplan-Meier method, and differences were assessed with the log-rank test. A multivariate analysis was performed using the Cox proportional hazards model. Two-sided *p*-values were computed, and the level of significance was set at 0.05. Statistical analyses were carried out using the SAS software package (release 9.4, SAS Institute, Cary, NC, USA).

## 3. Results

### Operative Procedures and Postoperative Outcomes

During the study period, 51 consecutive patients underwent the synchronous resection of PNETs and liver metastases. The median age was 54 years (range 27–80 years) with a gender ratio of 1.04 (26 men and 25 women) (Table 1). 

Most of the patients had PNET of the left pancreas (61%), while two had multifocal tumors. Functioning PNET with secretory syndrome was present in six patients [Glucagonoma (*n* = 2); Insulinoma (*n* = 1); Gastrinoma (*n* = 1), VIPoma (*n* = 2)]. Seven patients had neoadjuvant treatment including lanreotide (*n* = 4), taxol + bevacizumab (*n* = 1), gemcitabine (*n* = 1), carboplatine-VP16 (*n* = 1). The types of pancreatic resections included PD *n* = 16, DSP *n* = 32 and TP *n* = 3, and the synchronous resection of the mesentericoportal axis was performed in 16 patients, while arterial resection was performed in three. Seventeen patients also had a synchronous visceral resection. Synchronous hepatic resection was performed in 44 patients, while seven had exclusive radiofrequency ablation of LM. In twenty-one patients, resection was combined with RF ablation. Major hepatectomy (two right and two left) was performed synchronously in four patients, and the remaining liver resections were uni-, bi- or trisegmentectomies, and non-anatomical resection. Four patients with bilobar LMs underwent primary tumor resection and the clearance of the future liver remnant as a first stage of two-stage hepatectomy. Two patients failed to undergo the second stage because of postoperative portal vein thrombosis (*n* = 1) and death (*n* = 1). Postoperative mortality was recorded in one patient because of grade C pancreatic fistula eroding visceral vessels. The overall morbidity was 53%, with a major morbidity rate of 22%. Postoperative pancreatic fistula was recorded in 17.6% of the cases. Reoperation was needed in five patients postoperatively in the first 90 postoperative days. The causes of reoperation were hemorrhage due to grade C POPF, in order to derive the splenic vein (SV) after venous resection during PD with SV ligation due to sinistral portal hypertension, abdominal bleeding after DSP, intestinal occlusion, and marginal ulcers in patients with gastrinoma. The pathology showed a median tumor size of 50 mm (range 20–170 mm), with 38 patients having positive lymph nodes, with median numbers of 3 positive lymph nodes (range, 1–25). Most of the patients had G2 tumors (72%), while nine (18%) had G1 tumors and five (9%) had G3 tumors. The median Ki67 tumors were for G1, G2, and G3 of 1.8, 7 and 60, respectively (Table 2).

**Figure 1:**
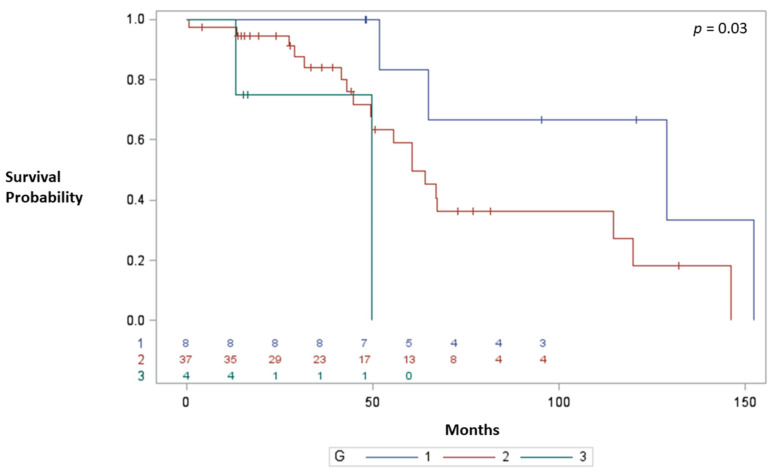
Median OS according to the tumor grade.

According to the type of resection, the patients undergoing right pancreatic resection had a statistically significant larger tumor size (*p* =0.03) and longer operations (*p* < 0.00001), and required more frequent venous resection (*p* = 0.04). There were no differences in the postoperative outcomes in terms of mortality and morbidity or reoperation according to the type of pancreatic resection. No differences were found in terms of the median OS rates between PD and DSP at 67.1 vs. 64.7 months. The median OS was 64.78 months (95% CI: 49.7 -119.8, from surgery), and the overall survival rates at 1, 3, 5, and 10 years were 97.9%, 86.2%, 61% and 29% respectively. During the follow-up, 23 patients recurred and 25 died. The median recurrence-free survival duration was 6.68 months (95% CI: 4.2–11.2). The median overall survival was longer in patients aged less than 65 years (67.0 months vs. 49.0 months; *p* = 0.08) and for G1 tumors (median OS 128 months, 5-year OS 83%) vs. G2 tumors (median OS 60.5 months, 5-year OS 58%) vs. G3 (median OS 49.7 months, 5-year OS 0%) (*p* = 0.03) (Figure 1).

Multivariate Cox analysis identified G3 tumours as the only prognostic factors (HR: 5.56; 95% CI: 0.91–9.60; *p* = 0.01) (Table 3).

## 4. Discussion

This study demonstrated that, in a specialized setting, the synchronous resection of PNETs with liver metastases can be performed with mortality and morbidity comparable to standard pancreatic resection. The oncological efficacy of this aggressive approach was associated with prolonged survival, especially in the presence of well-differentiated PNETs such as those reported in previous series [7,9,12]. The tumors’ grade differentiation, rather than the type of pancreatic resection and/or the localization of liver metastases, influences the oncologic outcomes. 

Combining liver and pancreatic resection carries the risk of cumulating the specific morbidities of the two separate operations. Cumulating the effect of a pancreatic and biliary fistula with liver failure can bring a mortality rate of up to 17%, as was demonstrated by a recent multicentric study on hepatopancreaticoduodenectomy [6]. In addition, because PNETs are most commonly discovered incidentally, patients present with soft pancreas and a not dilated pancreatic duct, which carries a higher risk of pancreatic fistulas. In fact, in the current study only four patients presented with jaundice and received a preoperative biliary stent. In the group of patients undergoing PD, the rate of postoperative clinically significant pancreatic fistula was 18% (3/16), with one postoperative death due to late bleeding. This patient—who received left-liver clearance and PD as a part of a two-stage procedure—had no biliary fistulas, but only POPF, which was the source of the lethal bleeding. This mortality arrived despite our policy of not combining major liver resection with PD. Kianmanesh et al. [8]—who reported a large series of two-stage procedures to treat primary NETs and LMs—reported no death, but only used this strategy in patients with left pancreatic tumors. Because of the expected long-term survival of well-differentiated metastatic PNETs, we believe that in patients with bilobar LMs and PNETs of the pancreatic head, synchronous resection should cautiously be planned. One strategy could be to separate the planned procedure into three stages, with primary tumor resection being performed as a last step in the presence of non-symptomatic PNET of the head, or a first step in the case of symptomatic tumors (jaundice, digestive symptoms, or venous contact). Postponing primary tumor resection could give priority to LMs which conditioned the overall survival of those patients while minimizing the risk of cumulating morbidities. Five patients required reoperation in this experience at a similar rate between left and right pancreatic resection. Only two were related to specific pancreatic complications, a rate which is in the range of that reported for pancreatic resections. 

The role of neoadjuvant chemotherapy in patients with LMs from PNETs remains to be evaluated [17]. Preoperative neoadjuvant chemotherapy has not been associated with tumor downstaging in locally advanced PNETs [18]**,** but it seems to improve the results of the resection of LMs from PNETs. Cloyd et al. [19] reported that the use of Fluorouracil, Doxorubicin, and Streptozocin (FAS) was associated with improved overall and disease-free survival when used in noeadjuvant settings in patients with LM from PNETs, compared with upfront resection. The use of neoadjuvant FAS in patients undergoing the synchronous resection of PNET with LMs (*n* = 46) was associated with improved median OS [97.3 months (95% CI 65.9–128.6) vs. 65.0 months (95% CI 28.1–101.9), *p* = 0.001] and RFS [24.8 months (95% CI 22.6–26.9) vs. 12.1 months (2.2–22.0), *p* = 0.003] compared to upfront resections. In our study, we did not have experience with this type of treatment because, in France, surgery is considered first in the presence of resectable liver disease. A prospective randomized study could be helpful to define the use of neoadjuvant FAS in the presence of PNETS with LMs, because the results of this treatment appear promising [19]. A weighted balance between safety and oncologic efficacy should guide surgeons when considering the combination of liver and pancreatic resection for metastatic PNETs. Liver resection for LMs of PNET has been associated with improved disease-specific survival when compared with unresected LMs. Klein et al. [9] reported that, among patients with LMs, the mortality rate was higher in those in whom liver resection was not possible than in patients who had liver resection (HR 9= 24.1–85.18; *p* = 0.049). In addition, patients who had liver resection had similar disease-specific survival to those without liver metastases (HR 0.84, 0.09 to 7.57; *p* = 0.877), and the tumor grade strongly influenced the overall survival. This study, however, presented a noteworthy selection bias. 

**Figure 2:**
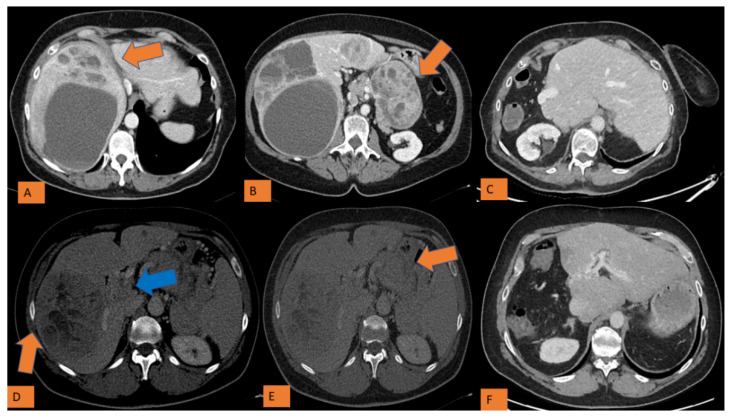
Two clinical cases of PNET of the left pancreas with bilobar LM treated by synchronous resection. Panel (**A**–**C**): a 54-year-old patient with well differentiated PNET of the left pancreas (KI-67 2%)(orange arrow) with bilobar LMs(orange arrow) who underwent lelft splenopancreatectomy with right hepatectomy and tumorectomy of the segment 2. Patient is alive without recurrence at 9 years. Panel (**D**–**F**): a 50-year-old patient with moderately differentiated PNET (KI-67 3%)(orange arrow) tumoral thrombosis of the splenic and portal vein thrombosis(blue arrow) and bilobar LMS(orange arrow). The patient underwent simultaneous lelft splenopancreatectomy with right hepatectomy and portal thrombectomy. Multiple Liver resection and RF ablation of LM into the left liver were performed three months later as a second step of a two-stage strategy. The patient is alive with liver recurrence under medical treatment 6 years later.

In the current study, we confirmed that in PNET tumor grade differentiation was a prognostic factor for the overall survival in presence of LMs [1,11,20]. Neither the tumor localization nor the extent of liver disease (even when resectable) had a greater impact than the tumor grade differentiation on the overall disease-specific survival. Well-differentiated PNETs showed longer survival rates (median OS 128 months, 5-year OS 83%), underlining the oncological efficacy in this patient population and the need to carefully evaluate the risk of surgical procedures in this population (Figure 2). 

The biology of the tumor should cautiously guide surgeons in the planning of aggressive surgery with potential morbidity, especially when scheduling PD for well-differentiated tumours of the pancreatic head in the presence of LMs. In this high-risk situation with soft pancreas and/or obese patients, separating the entire sequence in multiple separate stages could be an option. Additionally, these patients should be referred to tertiary centres for the minimization of postoperative morbidity.

Even if the results presented in the current study appeared favourable in terms of postoperative morbidity, the role of these extensive surgeries for PNETs in the presence of LMs remains to be debated, especially in the case of the PNET of the pancreatic head. The surgical resection of PNET of the left pancreas in the presence of non-resectable LMs seems to be associated with improved survival compared with non-operated patients [21]. While this seems to be related mostly to a selection bias, the surgical resection of the primary could be associated with fewer local complications, and could decrease the tumoral burden while leaving only liver disease to be treated either locally or by systemic therapy. While liver resection should be performed in the presence of diffuse, liver involvement also remains to be discussed. Recent advances in interventional radiology procedures including chemo and radioembolization seems to improve local control [1,2,3]. The benefit of liver resection should always to be balanced against the inherent risk of surgery. 

This study presents several limitations that deserve comments. First, its small size and the retrospective nature come with inherent limitations. A multicentric study exploring the policies and resection strategies of referral centres for PNETs with LMs could provide a further body of literature on this point. Second, the role of neoadjuvant and adjuvant treatment in this patient population with a high tumoral burden remain to be explored. It is likely that the use of neoadjuvant treatment could select the ideal patient population for these aggressive surgeries. Third, the optimal therapeutic sequence for the treatment of the primary PNET and LMs remains to be determined. Again, a multicentric study could help to identify the optimal treatment strategy for patients with PNET and LM. Fourth, we could not capture the exact denominator of this study regarding the patients that were seen at our institution and were judged not to be resectable. Finally, this study lacked a comparative analysis with other non-surgical treatment for LM, or a comparison with an exclusive control group treated by medical treatment. 

## 5. Conclusions

Simultaneous liver resection for metastatic pancreatic neuroendocrine tumors (PNET) can be performed safely with acceptable morbidity and mortality at tertiary centers. Well-differentiated PNETs showed longer survival, and might benefit the most from these extended surgeries. Optimal sequential surgical strategies for PNETs with LM and the use of neoadjuvant and adjuvant chemotherapy in this context remain to be further evaluated.

## Figures and Tables

**Table 1:** Clinical characteristics of the patient population (*n* = 51).

Age ^1^	54 (27–80)
F/M	25/26
Bilairy Stent	4 (8%)
Tumor localization	
Head	18 (35%)
Left pancreas	31 (61%)
Multifocal	2 (4%)
Chromogranine A (µ/L) ^1^	150 (19–15,500)
Functional	6 (11.7%)
Type of pancreatectomy	
Pancreaticoduodenectomy	16 (31.3%)
Splenopancreatectomy	32 (63%)
Total pancreatectomy	3 (6%)
Operative time ^1^	465 min (180–755)
Venous resection	16 (31.3%)
Arterial resection	3 (6%)
Liver resection	
Exclusive resection	23 (45%)
Exclusive radiofrequency ablation	7 (13.7%)
Resection and radiofrequency	21 (41.1%)
Associated visceral resection	17 (18,4%)
Transfusions	14 (27.4%)
Mortality	1 (2%)
Morbidity	27 (53%)
Major morbidity	11 (22%)
Pancreatic fistula	9 (17.6%)
Grade A	3
Grade B	5
Grade C	1
Reoperation	5 (9.8%)

^1^ = Data are expressed as the median (range).

**Table 2:** Pathology (*n* = 51).

Tumor size (mm) ^1^	50 (20–170)
No lymphnodes involvement (N0)	13
Lymphnodes invovled (Npos) ^1^	3.5 (1–25)
Lymphnodes harvested (Ntot) ^1^	23 (5–85)
Ki-67% ^1^	7% (1–80)
G1	9 (18%)
G2	37 (72%)
G3	5 (9%)

^1^ = Data are expressed as the median (range).

**Table 3:** Univariate and multivariate Cox regression analysis of the overall survival in relation to the clinicopathologic features.

Characteristics		Univariate Analysis			Multivariate Analysis		
	Median Survival Log-Rank (Months)	HR	95% CI	*p*	HR	95% CI	*p*
Age (years)							
<65 vs.	67.0						
>65 years	49.0	2.37	(0.89–6.25)	0.08			
Jaundice							
Yes	41.0						
Not	64.0	1.96	(0.64–5.86)	0.23			
Functional tumors							
Yes	64.0						
Not	64.7	0.84	(0.24–2.86)	0.78			
Tumor site							
Right	67.1						
Left	64.1	0.59	(0.25–1.42)	0.24			
Type of pancreatectomy							
PD	67.1						
DSP	64.7	0.69	(0.24–1.52)	0.28			
TP	35.2	5.26	(0.91–30.4)	0.06			
Venous Resection							
Yes	67.1						
No	64.7	0.76	(0.27–2.17)	0.60			
Number of LM							
2	128.8						
>2	64.0	2.25	(0.65–7.74)	0.19			
Radiofrequency							
ablation							
Yes	60.5						
Not	119.6	0.67	(0.48–1.34)	0.40			
Transfusion							
Yes	64.0						
Not	128.8	0.86	(0.32–2.34)	0.77			
Morbidity							
Yes	64.8						
No	66.8		(0.44–2.39)	0.94			
G (WHO 2010)							
G1	128.8						
G2	60.5	2.91	(0.85–10.04)	0.08			
G3	49.7	10.4	(1.56–69.4)	0.01	5.56	(0.91–9.60)	0.01
Lymphnode invasion							
N1	60.5						
N0	152.2	3.16	(1.21–8.22)	0.01			

## Data Availability

The data that support the findings of this study are available on request from the corresponding author. The data are not publicly available due to privacy or ethical restrictions.
